# Finger-Gesture Controlled Wheelchair with Enabling IoT

**DOI:** 10.3390/s22228716

**Published:** 2022-11-11

**Authors:** Muhammad Sheikh Sadi, Mohammed Alotaibi, Md. Repon Islam, Md. Saiful Islam, Tareq Alhmiedat, Zaid Bassfar

**Affiliations:** 1 Department of Computer Science and Engineering, Khulna University of Engineering & Technology, Khulna 9203, Bangladesh; 2 Institute of Information and Communication Technology, Bangladesh University of Engineering and Technology, Dhaka 1000, Bangladesh; 3 Faculty of Computers and Information Technology, University of Tabuk, Tabuk 71490, Saudi Arabia; 4 Industrial Innovation & Robotics Center, University of Tabuk, Tabuk 71490, Saudi Arabia

**Keywords:** computer vision, hand-gesture control, fall detection, obstacle avoidance, smart wheelchair

## Abstract

Modern wheelchairs, with advanced and robotic technologies, could not reach the life of millions of disabled people due to their high costs, technical limitations, and safety issues. This paper proposes a gesture-controlled smart wheelchair system with an IoT-enabled fall detection mechanism to overcome these problems. It can recognize gestures using Convolutional Neural Network (CNN) model along with computer vision algorithms and can control the wheelchair automatically by utilizing these gestures. It maintains the safety of the users by performing fall detection with IoT-based emergency messaging systems. The development cost of the overall system is cheap and is lesser than USD 300. Hence, it is expected that the proposed smart wheelchair should be affordable, safe, and helpful to physically disordered people in their independent mobility.

## 1. Introduction

Every human life deserves the free movement of its natural capabilities. But, due to disabilities and/or other unavoidable circumstances, many of us are unable to navigate safely or to do regular tasks independently. In health issues, disability refers to the inability for doing any sort of task [1]. The rate of disability is increasing sharply. People who are suffering from paralysis [2]; fatal injuries in legs, and hemiplegia [3]; different forms of neural and brain injuries; losing the capability of walking or moving on their own, need a supporting device like a wheelchair [4]. There are more than 75 million people with disabilities in the world who need a wheelchair, approximately 1% of the total population [5].

The wheelchair is raising the demand for people with disabilities to make their life easier [6]. Manual wheelchairs mitigating this necessity for many decades [7]. However, for paralyzed or people with severe disabilities, it is very difficult to drive a wheelchair manually. Hence, they need human assistance for their safe navigation. Again, the use of a manual wheelchair for a longer time might affect the users’ health and muscles [8]. Hence, power wheelchairs were introduced to minimize this problem [9]. But, in the case of many dexterities inhibited patients, who have very low energy and lower control of their hands, operating a traditional joystick is very difficult.

Different methods and systems have been developed using robotics [10], sensors, computer vision [11], brain-computer Interfacing [12], and human-machine interaction [13] to help people with disabilities using different technologies. The fusion of those technologies builds the Smart Wheelchair [14]. Recently, Computer Vision along with sensor technology and Machine Learning (ML) models are contributing significantly to the context of a smart wheelchair due to the rising capabilities of processing devices.

Though joystick operation is more accurate and has a higher level of control, the gesture is an easy way to control wheelchair operations for people with disabilities who have difficulties with typical joystick operations [15]. However, commercially available joysticks have relatively higher prices. From the practical point of view, it is found that it needs more energy and needs more hand and/or finger movements to operate the joystick [16]. Moreover, the joystick operations are complex. Several works have been done based on the gestures and movements of various organs, i.e., hand gestures, head movements, and eye gaze detection using RGB and Depth cameras. Although Depth cameras improve the results a bit by giving depth information about the environment, they are not cost-effective. Whereas, RGB cameras are cost-effective though there are some limitations in using these cameras due to the lighting of the environment and complex background situations [17]. Our research has focused on using RGB cameras for a cost-effective solution with the optimization of minimizing their limitations.

This paper presents a smart cost-effective wheelchair system that could be controlled using natural finger gestures and movements. Users need only to move their fingers on top of the wheelchair handles to perform any wheelchair operation. It requires less energy and simple operations. The proposed system uses a simple RGB camera (1 Mega Pixel, 30 FPS, and USB cable connection) to detect movements and gestures and there is no need for joystick operations or external sensor wiring.

Additionally, the user feels comfortable with finger movement as it is easier to train the operating methodology among the novice participants. The system is controlled in real-time by a Raspberry Pi 4 Model B (1 GB RAM and 1.5 GHz of CPU frequency), which is a low-cost minicomputer with a suitable configuration, to serve the purpose efficiently. The same RGB camera can be used for multiple control mechanisms (i.e., eye gesture, facial expression, or head movement-based control; IoT-enabled distant patient monitoring). The proposed system also includes obstacle detection [18,19], and fall detection [20], with IoT-enabled emergency SMS alerts to ensure the safety of the users.

People who have difficulties with existing wheelchairs, and/or who prefer to operate the wheelchair using natural finger gestures with less effort are the target user in this work. At the production level, it can be included a multimodal control system (i.e., on-demand head/eye gesture control and remote control with patient monitoring utilizing the same RGB camera). This proposed smart wheelchair can be available at a low and reasonable price to make it accessible to the mass people, who are less capable to purchase a power wheelchair. Hence, the need for such a system is from both users and caregivers.

The gesture recognition process consists of several stages which are outlined shortly as follows.

- Raw hand pictures are taken by the RGB camera from the top position.
- Preprocessing and background subtraction are done on those frames, YCrCb human skin color segmentation [21] and Haar Cascade Palm detector [22] have been used to detect the hand region.
- The hand region is then tracked continuously for better optimization rather than detecting on every frame.
- The gesture is detected by feeding the hand region into the pre-trained CNN model (with three hand gestures) with maximum probability.

The three gestures that are considered in this paper represent three modes: Drive, Stop, and Horn. In driving mode, the direction of a specific finger on the pad placed over the wheelchair handle is tracked and wheelchair movement is controlled accordingly. Stop mode is applied to stop the movement of the wheelchair, and Horn mode is applied to alarm when there is any obstacle.

The organization of the remaining sections is as follows. Gesture-based wheelchair control systems, which are recently developed, are described in Section 2. After that, Section 3 describes the methodology of the whole gesture recognition process along with the necessary hardware and sensor implementation. Next, Section 4 measures the performance of training, validation, and testing of the model with the overall system and compares the results with other similar recently developed systems. Finally, the summarization, limitations, and potential directions for future research are described in Section 5.

## 2. Related Work

Researchers and developers are working continuously for the significant improvement of the safety and facilities of smart wheelchairs. Several smart wheelchair systems have been developed with various control mechanisms in the last few years. For consistency, we have described some of these methods related to the proposed system.

For instance, a head movement tracking-based wheelchair control model has been proposed by Kutbi et al. [23]. In this work, an egocentric camera has been used to capture the pictures of the head. TI-TAN18CS modeled wheelchair, Arduino Mega was used as a command processor, and Robot OS (ROS) has been used as the framework. The system obtained a performance of about 85.7%. The cost of the system remains high and wearing an egocentric camera on the head is not user-friendly.

An eye-pupil tracking-based wheelchair movement system has been proposed by Tejonidhi et al. [24]. This system used a Philips microcontroller and Viola-Jones MATLAB algorithm for detecting the eyes from RGB images. However, the detection process is critical for real-time applications, and also the performance varies from 70% to 90%. Thus, it cannot be implemented in a real-life scenario.

Utaminingrum et al. developed a wheelchair-controlling mechanism by tracking a target object at the front of the wheelchair using RGB images [25]. The wheelchair movement control flows according to human detection using the HOG algorithm [26], interested human tracking using the CAMSHAFT algorithm [27], and movement detection. Target selection from multiple human objects is a complex and problematic scenario in this system, performance varies by around 80%, and needs to improve for real-life applications.

Furthermore, a multi-modal wheelchair control mechanism has been developed by Mahmud et al. [28]. This system used an accelerometer for head movement tracking, flex sensors glove for hand tracking, and an RGB camera with a modified VGG-8 model for eye gaze tracking along with raspberry pi. The performance varies around 90%. The limitations are that tracking sensors are needed to attach to the user’s body, and the eye gaze detection mechanisms are not user-friendly.

Desai et al. developed a control system for a wheelchair based on the eye’s iris movement [29]. In this proposed system, a user can control the navigation of their wheelchair by moving their iris in respective directions. MATLAB embedded programing language has been used for programming the model and the system response time needed 5 s longer. Again, the eye’s iris movement tracking might result in false positives sometimes due to subconscious movement of the eye in a real-life scenario. Thus, the system is unable to perform navigation operations in real-time and is hard to apply in a real-life scenario.

A hand gesture-based control mechanism for a power wheelchair has been proposed by Gao et al. [30]. An RGB-depth camera has been used for capturing the hand’s RGB and depth information. A high-configuration laptop PC has been used for detecting and tracking gestures. The drawback of this proposed system is that the background complexity of the environment affects the performance of this system severely. Also, the system is not user-friendly as the raising of a hand is mandatory for performing gestures, and the system cost is high.

In literature, Bhuyain et al. developed an Electrooculogram (EOG)-based wheelchair movement controlling system [31]. Eye blinks and movements have been captured using simple electrodes placed beside the eyes. They used a microcontroller and threshold-based algorithm and a locally developed power wheelchair for implementation purposes. Thus, the cost of the system is very low, but placing new electrodes beside the eyes each time is difficult.

## 3. The Proposed Methodology

**Figure 1:**
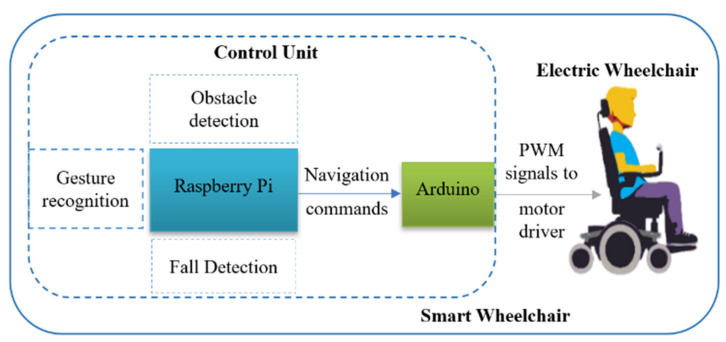
Simplified block diagram of the proposed smart wheelchair.

The design of the proposed system can be split into two parts. First, gesture recognition is designed with the aid of computer vision technology. Second, the hardware implementation, which consists of assembling different types of sensors and hardware control devices, is performed. Figure 1 shows the simplified block diagram of the proposed system that combines gesture recognition and hardware control.

There are two processing units in the system: Raspberry Pi 4 model B and Arduino Uno microcontroller. The Raspberry Pi is used as the main processing unit which performs gesture recognition and sends commands to Arduino. Here, Arduino is used for interfacing between the Raspberry Pi and two motor drivers for motors’ navigation. Arduino receives control commands based on the input gestures from the main processing unit (Raspberry Pi) through serial communication. Then it sends specific PWM signals toward motor drivers. In addition to wheelchair navigation such as forward, backward, left, right, and stop, our system is designed for obstacle detection using five Ultrasonic sensors, and fall detection using an accelerometer with IoT-based emergency SMS alert functionality.

### 3.1. Gesture Recognition

There are several stages in the Gesture Recognition process. Firstly, the detection of the hand palm has been done using Haar Cascade Classifier for Palm detection, as it works effectively with Haar features, a lightweight model for palm detection in real-time. Before that, the hand region has been roughly separated using The YCrCb color space with the human skin color range (0, 133, 77) to (235, 173, 127) along with essential preprocessing of the frame (frame resizing, noise reduction, and binarization) [32]. In the second stage, the Kernelized Correlation Filters (KCF) algorithm has been utilized to track the detected Region of Interest (ROI) which is the targeted hand region. KCF tracking algorithm works better for tracking the hand palm and finger rather than other tracking algorithms due to its computing mechanism. Thirdly, gesture recognition has been done using the previously trained finger gesture recognition model by resizing and feeding the frames’ ROI to predict the relevant finger gesture.

**Figure 2:**
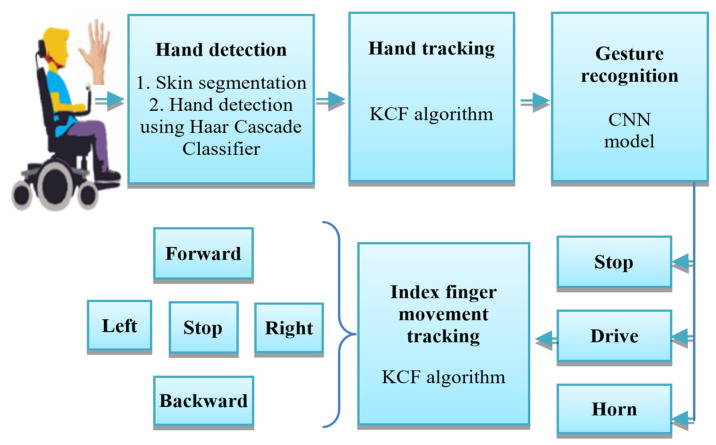
The overall scenario of the gesture recognition process.

Three gestures have been considered in this work: ‘Stop’, ‘Drive’, and ‘Horn’. In ‘Drive’ mode, a second KCF tracker has been utilized to track the movement of the index finger for detecting the four directional commands: Forward, Backward, Left, and Right. The overall scenario of the gesture recognition process has been discussed in the following subsection and shown in Figure 2.

#### 3.1.1. Hand Detection and Tracking Method

This is the first stage of the overall hand detection method. The initial captured frame has been resized into 160 × 240 pixels. The process of segmenting the human skin area is known as skin segmentation. The YCrCb color space with a natural human color range from (0, 133, 77) to (235, 173, 127) has been utilized for the segmentation of skin color [33]. The resized frame has been further processed to separate the hand region roughly from the background using this human skin segmentation method. The blurring, opening, and closing effects of basic image processing have been applied for noise reduction. The binarization of the frame has been done using the dilation effect to make the image black and white (binary image).

**Figure 3:**
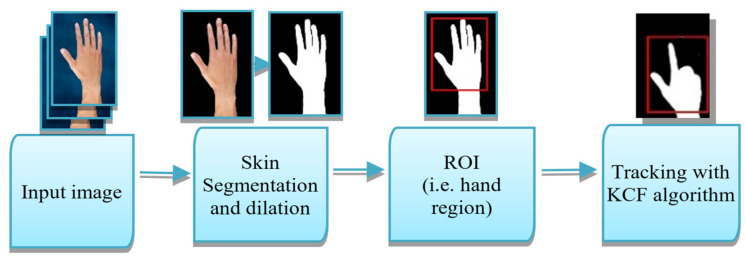
Hand region detection and tracking process.

Haar cascade classifier is a feature-based object detector that can effectively detect various human organs: faces, eyes, and hands, as well as other non-human objects. It utilizes machine learning techniques. Several negative and positive images are needed to feed for training the initial model [34]. This classifier has been used to effectively detect and obtain the bounding area of the actual hand region (ROI). A suitable hand region is identified with the highest palm width and height. We ignored many other small hand regions that are returned by the classifier as false positives. This process requires lesser than a second to identify the actual hand region successfully. The KCF tracking algorithm has been initialized to that bounding area of the frame for tracking the hand region. As detection is a resource-hungry and time-consuming process, especially in the case of a lower computational powered device like Raspberry Pi, tracking the region is an effective possible solution for obtaining real-time performance rather than detecting the hand palm whenever a new frame arrives. All steps of the detection and tracking process are shown in Figure 3.

#### 3.1.2. Gesture Recognition Using 2D CNN

**Figure 4:**
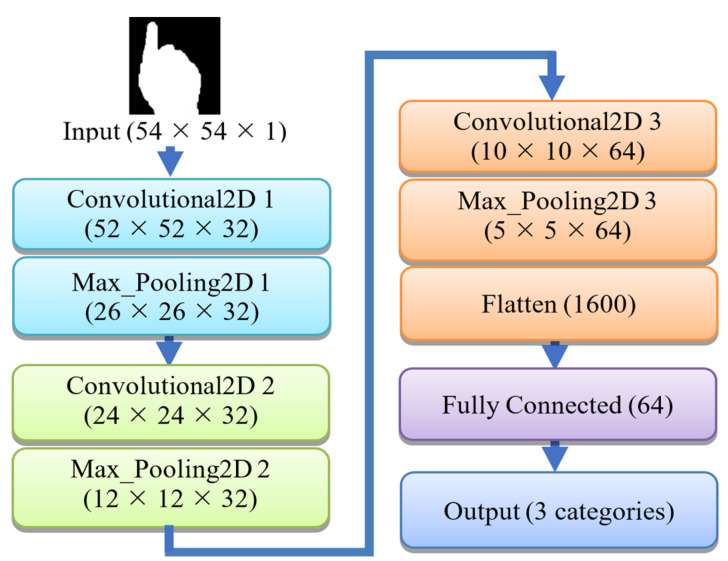
Architectural layers of the proposed CNN Model.

This is the most crucial stage of the overall gesture recognition process. The cropped hand region has been resized into a size of 54 × 54 for real-time processing of the frame. The resized binary hand-cropped image has been passed into the pre-trained hand gesture recognition model based on a 2D CNN. The proposed 2D CNN model contains three convolutional and three max-pooling layers. All calculations and updates are done on these layers based on the image pixels’ value. All the calculated features are flattened into a vector of size 1600 in the flatten layer, and those are recalculated in the fully connected or dense layers of size 64. Finally, the probability for each of the three classes has been predicted in the output layer. The architecture of the proposed 2D CNN model is shown in Figure 4.

The initial input frame size is 54 × 54 with a single binary channel. But, in the convolutional layer it reduces to 52 × 52 with 32 feature maps because any padding method, i.e., zero-padding is not utilized. Rectified Linear Unit (ReLU) activation function has been applied to each convolutional layer. The max-pooling layer has a kernel of size 2 × 2. It pools the maximum value from the 2 × 2 kernel values. Thus, the output of this layer has a size of half of the size of the input image in this layer. For example, if the input image is 52 × 52, then the output of this layer will have a size of 26 × 26.

Similarly, in the second convolutional layer, the image has reduced to 24 × 24, and after max-pooling it reduces to 12 × 12. After the third convolutional and max-pooling layer, the final dense layer received an input size of 5 × 5 with 64 feature maps. The flatten layer makes the output of the last pooling layer into a single vector of size 1600. A fully connected layer with 64 neurons as input parameters has been utilized to turn the 1600 features into 64.

Finally, after measuring the network testing, the integration of ReLU, cross-entropy, and softmax activation is done with all parameters. The proposed model works efficiently with a few training data and the training images have lesser dependencies on the environment, background, or person because the training data is finally binarized and generalized.

**Figure 5:**
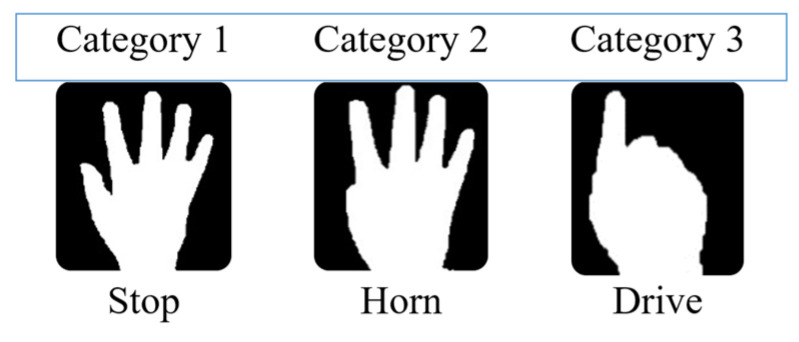
Classes of the predicted gestures that are recognized for wheelchair control.

Each layer has been randomly initialized with weight parameters for the network training. Then, for estimating the result of the output, the “softmax” activation function has been utilized. Afterward, for calculating the error of the output, the “cross-entropy” has been used as the loss function. The final output array gives the probability of the three pre-defined gestures. Among them, the gesture with maximum probability has been selected for further actions. The three considered gestures are shown in Figure 5.

#### 3.1.3. Wheelchair Control

In our previous paper [15], six different categories of gestures were used for controlling the entire wheelchair operations without considering any finger tracking. In this work, feasibility study has been performed with different users for evaluating the uses of different gestures and easy control of the wheelchair in real-life scenarios. It is observed from this study that a single finger movement is much easier rather than moving all fingers to form a corresponding gesture for navigating the wheelchair in any direction. As a result, single finger movement has been tracked in this paper.

**Figure 6:**
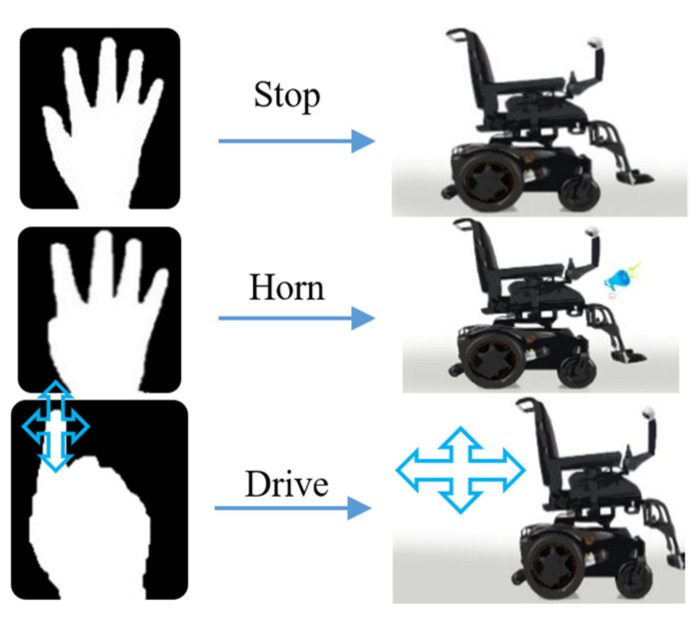
Operation of the proposed system.

**Figure 7:**
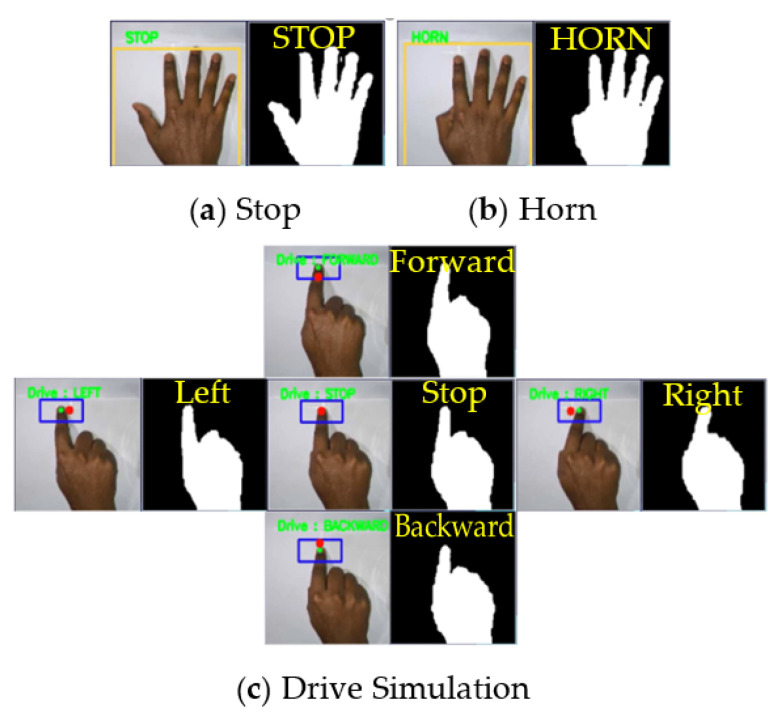
Different gestures and movements simulation (**a**) Stop; (**b**) Horn; (**c**) Drive Simulation.

Three types of hand gestures have been considered for three basic control operations named Stop, Horn, and Drive. The highest probable gesture from the probability array given by the CNN model has been considered. Stop gesture is used as the initial gesture to stop the wheelchair at any time or condition. The horn gesture is used for signaling the environment. When the Drive gesture is shown, the system goes to drive mode and the wheelchair movement operations are done in this mode. The single fingertip is identified by calculating the area in the upper portion of the palm where the tip can be located and then the actual tip is found using contour finding algorithm in this area. Finally, the single finger’s initial position is stored, and then the movement of the finger is measured along x and y coordinates. A minimum amount of distance (predefined threshold is selected upon experimentation, i.e., 10 pixels for x or y direction) must be reached at a particular direction for performing any movement accordingly for recognizing the four directional movements, i.e., left, right, forward, and backward with initial stop positions. The basic controlling gestures are shown in Figure 6, and the basic gestures and directional movement operations are shown in Figure 7. The user can use gestures to communicate with others out of the limited region of this gesture recognition process, or use gesture rather than any directional command gesture (i.e., left, right, forward, backward). The wheelchair will automatically stop if any directional command gesture is not recognized in three sequential image frames.

### 3.2. Hardware Implementation

The configuration of the system is constructed with two processing units: one Raspberry Pi module, and one Arduino module; along with other components such as an RGB camera (for image capturing), 12 V rechargeable Lead Acid battery (for power supply), two DC motors having a power rating of 250 W, two motor drivers, Ultrasonic sensors, and accelerometer for giving functionalities like obstacle detection and fall detection with IoT operations.

#### 3.2.1. Processing Units

In the proposed system, two processing units Raspberry Pi and Arduino are used to execute the required functionality. They are connected via Arduino USB data cable for serial communication with a particular port address.

Raspberry Pi 4 with 1 GB of RAM works as a main processing unit. Its raw input voltage is 5 V with 3 A, and the clock frequency is 1.5 GHz. For powering up this unit, a DC-DC buck converter is used to convert the 12 V from the supply battery to 5.4 V with a maximum 3 A output current, which is needed for the Raspberry Pi in overclocking mode. Thus, CPU speed goes up to 2.0 GHz and provides faster real-time processing. It generates the control commands by performing gesture recognition using a pre-built 2D CNN model and finger tracking using the KCF algorithm. Python 3 programming language has been used for program development.

It can process accelerometer and ultrasonic sensors’ data for obstacle avoidance and fall detection as well. For obstacle avoidance, the ultrasonic sensors’ (five sensors are mounted in four different directions) data have been read continuously. If the distance toward any direction becomes lower than a specified threshold (i.e., detect the obstacle at the front if the distance in that direction is lesser than 50 cm). The distances toward the obstacles are marked by red color in the GUI and notified the users by generating an alarm signal. Necessary actions (i.e., stop the wheelchair if the obstacle is in the way of the driving direction) can be taken based on the responses. For IoT-enabled fall detection, we have continuously read the motion sensors’ data and listened to any sudden changes due to the occurrence of falls and used threshold (based on values shown by the sensors for events like sudden fall) to detect any fall. If any fall occurs, then an emergency alert signal is generated. An SMS server has been built by developing an Android application using Google Firebase. Fall and emergency information (including the preconfigured emergency contact numbers and fall types i.e., left, right, forward fall, or backward fall) are sent to that SMS server if the internet is available on the Raspberry Pi. When this information is loaded on the server, Emergency SMS is sent with the fall information to the predefined emergency contact numbers. An Android device with a SIM card (having enough balance) is connected to the SMS server for this communication purpose. Moreover, an SMS server API can be directly integrated with Google cloud to fulfill this purpose. This information can be shown to multiple domains through multiple mediums (i.e., using an android device, web app, etc.). Other health-related sensors can be incorporated according to wheelchair patients’ requirements.

**Figure 8:**
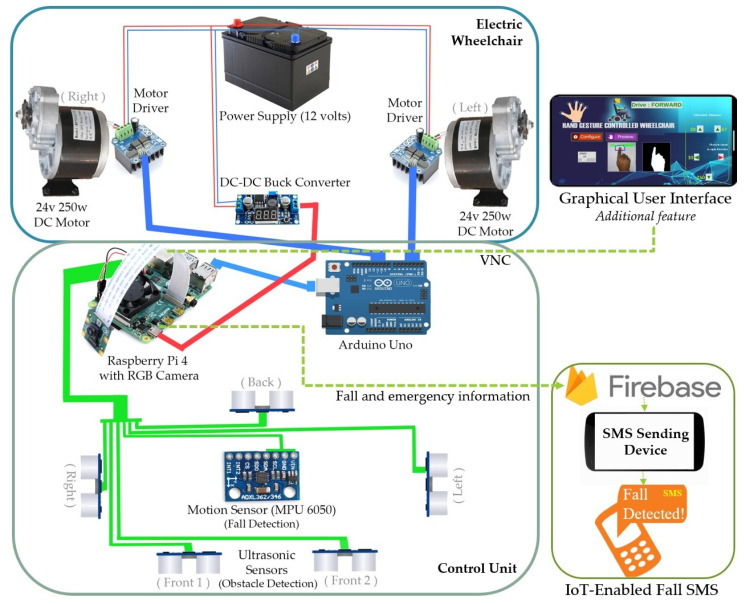
Interfacing of different components of the proposed smart wheelchair system.

In this system, Arduino Uno has been used for interfacing between Raspberry Pi and the BTS7960 motor driver. It is connected to the Raspberry Pi via USB cable and powered up by Raspberry Pi. Raspberry Pi has a single usable PWM pin and is also troublesome in many cases. The motor drivers need to be connected with four respective PWM pins. As Arduino has 6 digital PWM outputs, it is easy to interface the motor driver with Arduino instead of Raspberry Pi (have only a single PWM pin). The overall connections between the different components that are used in the proposed system are shown in Figure 8.

#### 3.2.2. Electric Wheelchair

**Figure 9:**
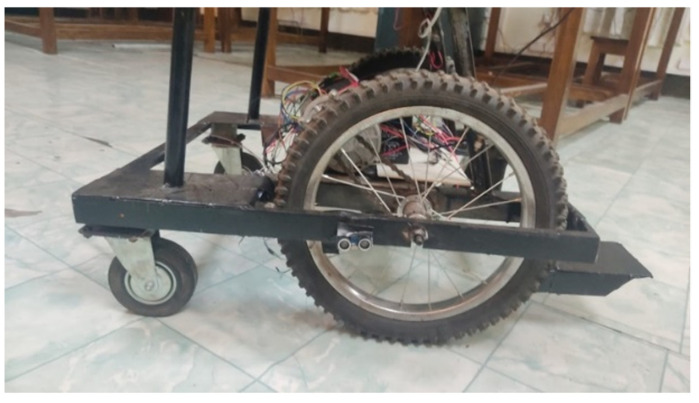
The base structure of the proposed smart wheelchair.

Without using of pre-built power wheelchair, a prototype of an electric power wheelchair is developed for the low-cost implementation with gesture control. However, it is constructed by two motors, two motor drivers, a battery, and other control circuits. Figure 9 shows the base structure with the necessary hardware components of the proposed smart wheelchair.

**Figure 10:**
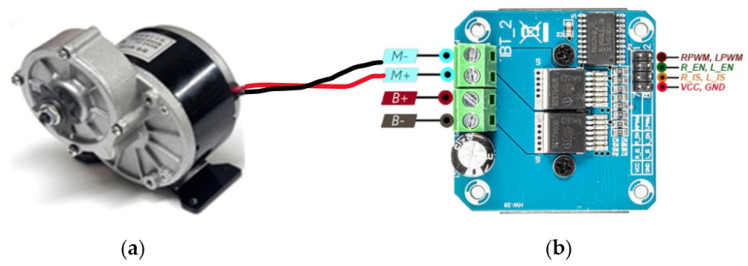
Interfacing of (**a**) Used motor and (**b**) motor driver.

One of the major components of an electric wheelchair is the motor that should be capable of carrying certain weights and fit system requirements. In our system architecture, the MY1016Z2 motor is used as shown in Figure 10a. It operates in 24 V, and 13.4 Amps and the power rating is 250 W (0.33 Horsepower). Its roller width and diameter are 0.16 inches and 0.3 inches respectively. This motor is suitable for rotating in two directions: Clockwise or Counterclockwise whose rated load RPM is 400, and no-load RPM is 3300.

BTS7960 is the model number of the used motor driver in our system which is shown in Figure 10b. The specification of this driver is suitable to meet our requirements. It has a half-bridge control circuit, which is required for the DC motor for rotating forward and backward. Besides these, it can operate in 24 V, 43 A max; capable of PWM (Pulse Width Modulation) up to 25 kHz; and suitable for handling high power motor.

**Figure 11:**
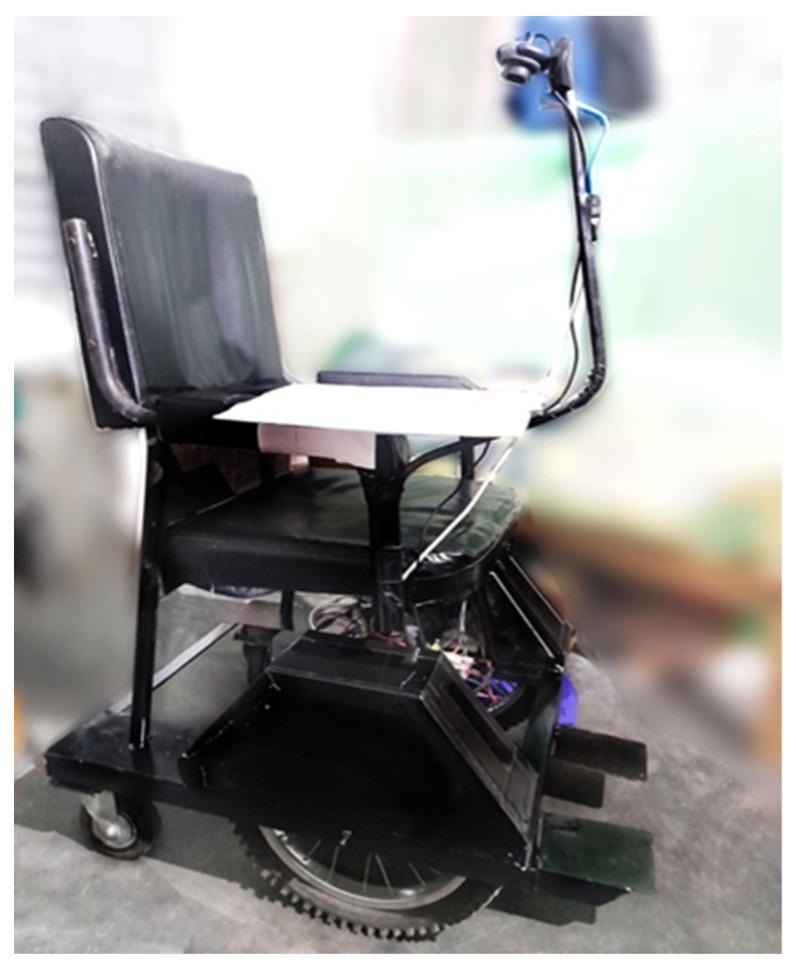
Completed structure of the proposed smart wheelchair system.

The control circuitry is made up of a combination of two motor-driver, one Arduino-Uno processing unit, and their connection with the DC power supply. Motor driver BTS7960 has an effective H-bridge control circuit, which gives more convenience than the traditional four relay control circuit. Each motor driver has 8 control pins including +5 V Vcc and GND; three pins of them are R_EN, RPWM, R_IS that are involved in forwarding rotation, and the other three L_EN, LPWM, L_IS are involved in the backward rotation. These 8 control pins are getting signals from connected Arduino-Uno. Arduino is connected with Raspberry pi for getting signals based on certain gestures. Hence, bypassing specific PWM values through Raspberry pi, the wheelchair user can operate the motor’s speed and rotation. The complete model of the proposed smart wheelchair system is shown in Figure 11.

## 4. Experimental Analysis

The overall performance and comparison of the proposed smart wheelchair are analyzed in this section. The three gestures that are recognized through the CNN model and the four directional finger movements are taken into consideration for performance analysis. For training and validation of the proposed 2D CNN model, a total of 750 hand gesture images have been collected for the three mentioned gestures. Different lighting and environmental conditions were present when those images were captured. Any image processing technique has not been utilized to simulate different lighting conditions and noises for generating training images. Each gesture contains 200 training images and 50 validation images. As the images are preprocessed carefully and converted to a binary channel, the images are more generalized. Hence, this small number of training images could predict the best gestures among the three classes. The training of the model has been done in 20 epochs, where each epoch contains 2000 steps. It is mentionable that the training has been done in a medium configuration laptop rather than in Raspberry Pi as it is a resource-hungry but one-time process. The system has been trained to make more reliable and accurate decisions in different conditions (i.e., background complexity and occlusion) with CNN, and make the model lighter and faster by using Tensor-flow lite (as backend) so that the system can run in real-time with a low-computational powered device.

Accuracy and loss of the training, validation, and testing phases have been considered as evaluation matrices. Table 1 shows the performance measurement of this system. Training and validation accuracies are 96.85% and 99.42%, and losses are 0.0914 and 0.00035 respectively. These accuracy and loss denote the performance during the training and validation of the three basic gestures, i.e., Stop, Horn, and Drive.

**Figure 12:**
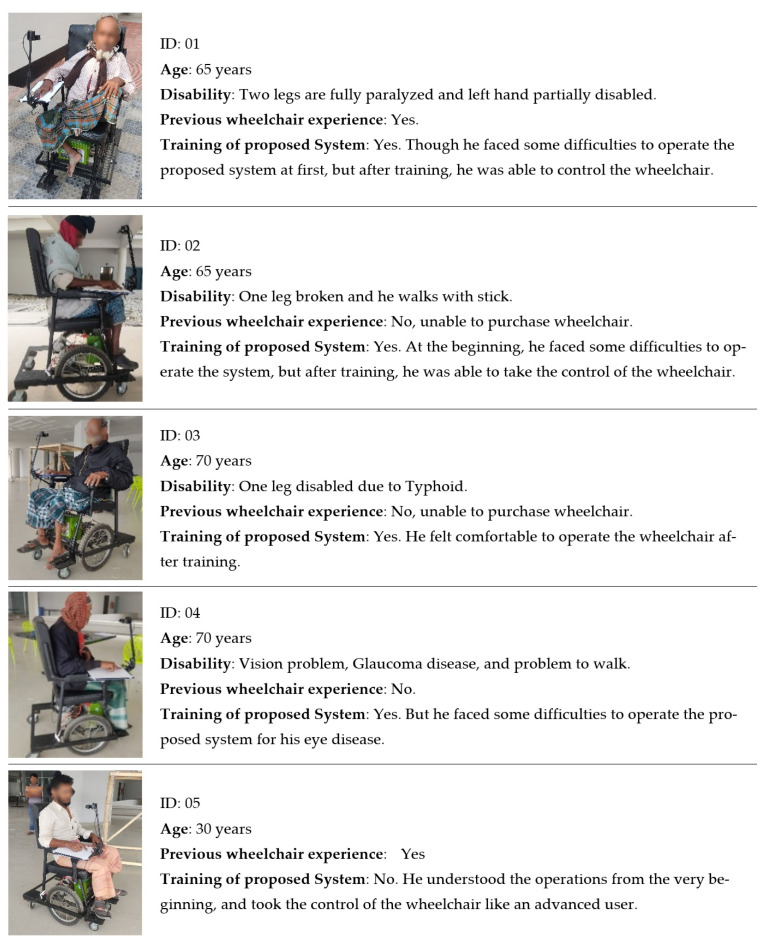
User trials of the proposed hand gesture-controlled smart wheelchair.

A number of people with various disabilities have participated in the testing phase of this system. The majority of them are elderly people aged from 65 to 70 years, having disabilities in legs and other organs. Figure 12 shows the proposed smart wheelchair with different people with disabilities. The experiment was conducted both in indoor and outdoor environments on various surfaces. The four movement control tasks have been performed by the participants at a pre-configured speed. The outcome shows that everyone is capable to control the wheelchair. Some users faced little difficulties at the beginning, but they were able to take the control of the wheelchair after one or two trials. Others felt comfort operating the wheelchair from the very beginning. From the practical point of view, it is experienced that there is negligible effect on vibration or on the changes of surfaces. A wheelchair handle/arm is used to stabilize the hand of the user while driving the wheelchair.

The wheelchair operations are based on the correct recognition of the gestures. Different users have been participated in the performance test and performed different gestures in different environmental conditions to test the feasibility of the proposed system. To quantify the results, responses with its original gesture have been recorded manually for each gesture and command. This action is performed 100 times for each gesture and a total of 700 gestures are captured from all participants. the responses are recorded as a confusion matrix as shown in Table 2. Gesture recognition steps perform very well except a little bit of confusion arises between Horn and Drive mode when the fingers are too close to each other and noisy during Horn mode. Thus, a few numbers of horn gestures are falsely detected as drive.

The derived accuracy is measured based on the correct detection of the finger gestures by the proposed system. We have counted the responses to the gestures in different angles and several lighting conditions. During finger movement tracking in driving mode, sometimes the tracker was unable to find the location of the fingers when they moved too rapidly due to the limitations of the hardware (e.g., the used camera had a low frame rate and could not capture the finger movement smoothly), and the system went into stop condition. Despite those situations, it achieves around 97.14% of testing accuracy. The safety of the proposed smart wheelchair was assessed by evaluating the ability to detect obstacles and falls, and after several testing, it shows that those feature work correctly.

Table 3 shows the comparison among the proposed system and the recently developed similar work. Gao et al. [30] proposed a hand gesture recognition-based system that uses a Microsoft Kinect Depth camera which is a costlier item. Again, the performance severely decreases when background complexity arises. It is also not user-friendly as the hand needs to be raised for performing any gestures. Oliver et al. developed a control system with joystick manipulation using accelerometer hand movement detection [35]. But it requires hand band wearing and the cost remains medium as the wheelchair (having basic power) prices are still high in the markets. Our proposed system has hand gesture and finger tracking mechanisms, which is easier and more user-friendly as it has IoT-based fall detection with an emergency SMS alert system and obstacle avoidance safety mechanisms. The overall estimated development cost of the proposed smart wheelchair has been shown in Table 4. There is a huge cut down in the price and can be available for lesser than $300 at the mass production level, and the performance is also feasible for real-time application.

**Figure 13:**
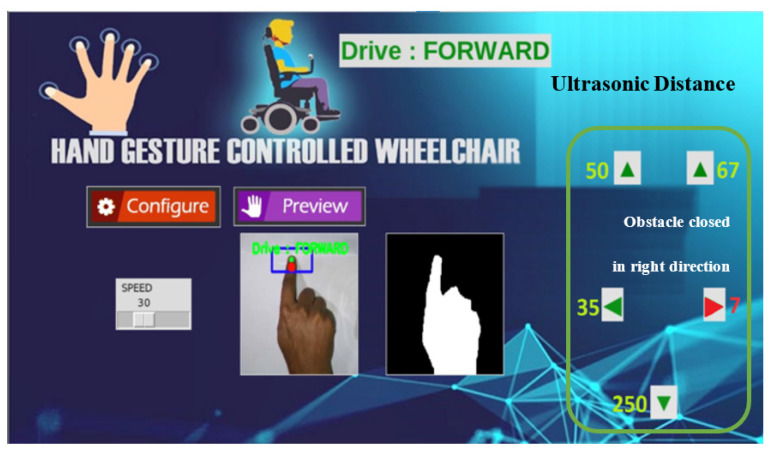
Graphical User Interface of the proposed system.

A Graphical User Interface (GUI) has been developed for the starting configuration (i.e., Speed, gesture preview hide/show) and viewing the live interactions with the proposed system as shown in Figure 13. The speed of the wheels can be configured using the speed slider in the UI, and the distance between the five ultrasonic sensors and obstacles can be shown in this UI with a red alert in centimeters (cm). During the testing, it is experienced that checking the GUI while driving is not needed. Moreover, it can help a user with self-training.

## 5. Conclusions

A cost-effective gesture-controlled smart wheelchair system is proposed in this paper. This system ensures comfortable navigation and safety for wheelchair users with extreme disabilities. As basic safety features, the system includes obstacle detection, and fall detection with an emergency messaging system (by developing an SMS server using Google Firebase) which sends fall and emergency information on the server and the server sends SMS to the predefined contact numbers. The system achieves a testing accuracy of 97.14% after experimenting with 700 gestures from different users. Detecting the hand region in extreme lighting conditions and colour variations, especially for static skin colors, is still challenging. In the future, this research can be extended to mitigate this problem. Moreover, the system can be enhanced to make it more robust by extending robotic, and advanced IoT-enabled features at optimal cost.

## Figures and Tables

**Table 1:** Performance of the proposed Hand Gesture-Controlled Wheelchair system.

Performance Measure	Training	Validation	Testing
Accuracy	96.85%	99.42%	97.14%
Loss	0.0914	0.00035	

**Table 2:** Confusion matrix for the testing of the proposed smart wheelchair system.

	Prediction	Stop	Horn	Drive Forward	Drive Backward	Drive Stop	Drive Left	Drive Right
Performed								
Stop		100	0	0	0	0	0	0
Horn		0	97	0	0	3	0	0
Drive Forward		0	0	97	0	3	0	0
Drive Backward		0	0	0	95	5	0	0
Drive Stop		0	0	0	0	100	0	0
Drive Left		0	0	0	0	2	98	0
Drive Right		0	0	0	0	7	0	93

**Table 3:** The comparison among proposed system and the similar work.

Proposed System	Features				
	Functionality	Equipment	Requirements and Limitations	Cost	Success Rate
Gao et al. [30]	Hand gesture recognition	Microsoft Kinect Camera, high configuration laptop	Require hand raising, background complexity	High	10–100%, depended on background complexity
Oliver et al. [35]	Hand movement detection	Accelerometer, Joystick Manipulator	Require hand band wearing	Medium	Actual accuracy is not shown
Our System	Hand gesture, fall detection, obstacle avoidance	RGB Camera, Raspberry Pi, sensors	Require only fingers’ movement	Low	97.14%

**Table 4:** Estimated cost of the developed smart wheelchair.

Items	Estimated Cost (in Dollars)
Electric Wheelchair Development 2 Motors with driver Physical Structure Power Supply	$ 100 $ 60 $ 50 _____________ $210
Control Unit Integration Raspberry Pi 4 (1 GB RAM) RGB Camera Arduino and sensors	$ 60 $ 15 $ 15 _____________ $90
Total	$300

## Data Availability

Not applicable.
